# Diabetes and infection: review of the epidemiology, mechanisms and principles of treatment

**DOI:** 10.1007/s00125-024-06102-x

**Published:** 2024-02-20

**Authors:** Richard I. G. Holt, Clive S. Cockram, Ronald C. W. Ma, Andrea O. Y. Luk

**Affiliations:** 1 Human Development and Health, Faculty of Medicine, University of Southampton, Southampton, UK; 2 Southampton National Institute for Health Research Biomedical Research Centre, University Hospital Southampton NHS Foundation Trust, Southampton, UK; 3 Department of Medicine and Therapeutics, The Chinese University of Hong Kong, Hong Kong Special Administrative Region, People’s Republic of China; 4 Laboratory for Molecular Epidemiology in Diabetes, Li Ka Shing Institute of Health Sciences, The Chinese University of Hong Kong, Hong Kong Special Administrative Region, People’s Republic of China; 5 Hong Kong Institute of Diabetes and Obesity, The Chinese University of Hong Kong, Hong Kong Special Administrative Region, People’s Republic of China

**Keywords:** Antimicrobials, Bacteria, Diabetes, Epidemiology, Infection, Pathogenesis, Review, Virus

## Abstract

An association between diabetes and infection has been recognised for many years, with infection being an important cause of death and morbidity in people with diabetes. The COVID-19 pandemic has re-kindled an interest in the complex relationship between diabetes and infection. Some infections occur almost exclusively in people with diabetes, often with high mortality rates without early diagnosis and treatment. However, more commonly, diabetes is a complicating factor in many infections. A reciprocal relationship occurs whereby certain infections and their treatments may also increase the risk of diabetes. People with diabetes have a 1.5- to 4-fold increased risk of infection. The risks are the most pronounced for kidney infection, osteomyelitis and foot infection, but are also increased for pneumonia, influenza, tuberculosis, skin infection and general sepsis. Outcomes from infection are worse in people with diabetes, with the most notable example being a twofold higher rate of death from COVID-19. Hyperglycaemia has deleterious effects on the immune response. Vascular insufficiency and neuropathy, together with altered skin, mucosal and gut microbial colonisation, contribute to the increased risk of infection. Vaccination is important in people with diabetes although the efficacy of certain immunisations may be compromised, particularly in the presence of hyperglycaemia. The principles of treatment largely follow those of the general population with certain notable exceptions.

**Graphical Abstract:**

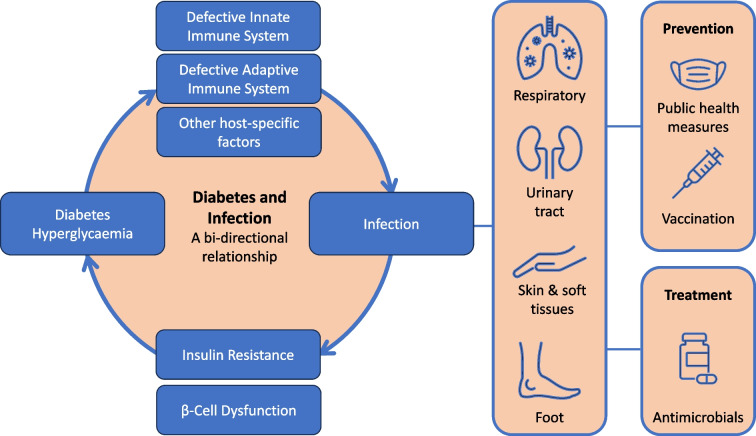

**Supplementary Information:**

The online version contains a slideset of the figures for download available at 10.1007/s00125-024-06102-x.


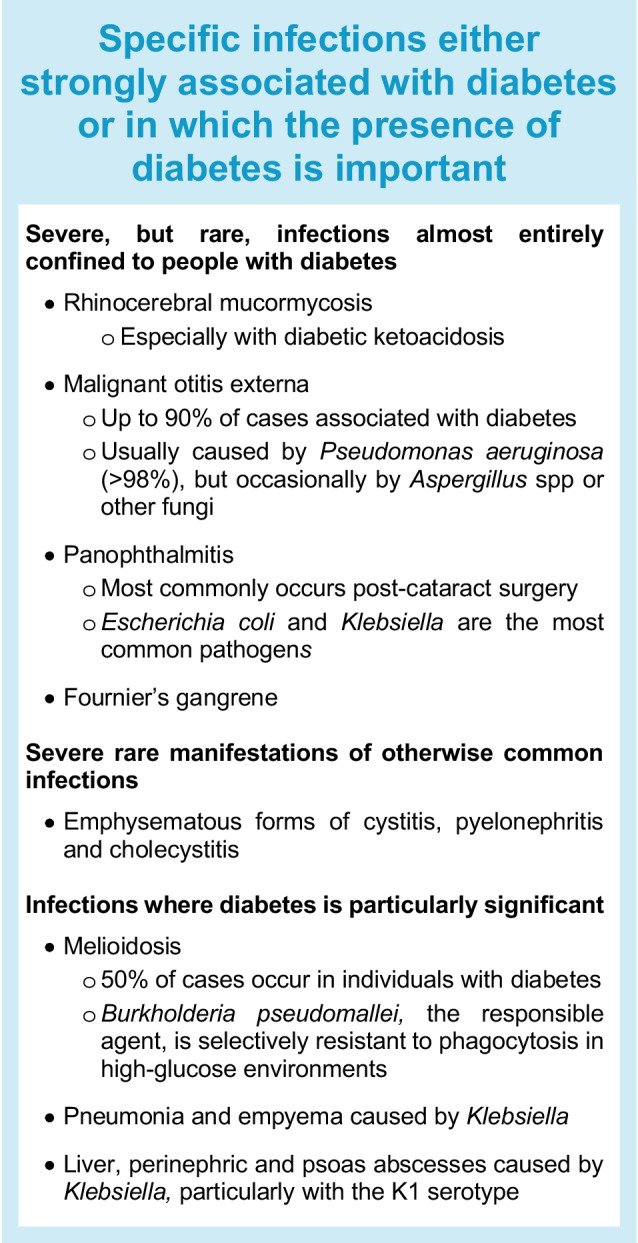


## Introduction

Historically, infections have been an important cause of death and morbidity in people with diabetes [1, 2]. Although this remains the case, particularly in low- and middle-income countries where infections are commonly presenting features of previously undiagnosed diabetes [3], infection has been an under-studied complication of diabetes. People with diabetes develop infections more often than the general population and the course of infection is more complicated [4–8]. The COVID-19 pandemic has re-kindled interest in the complex relationship between diabetes and infection, following observations that people with diabetes are more likely to progress to severe COVID-19 disease and die than those without diabetes [9].

Some severe infections occur predominantly in people with diabetes [10–13]. These tend to be uncommon but convey high mortality rates without early diagnosis and treatment (see Text box). Conversely, diabetes is more often a complicating factor in common infections, where the clinical course may be heterogeneous and affected by factors including glycaemic levels (both recent and longer term), diabetes-related complications and obesity [4–8].

Healthcare professionals should be aware of the relationships between diabetes and infection to watch out for serious manifestations of common infections. This review aims to describe the bi-directional relationship between diabetes and infection, before examining the mechanisms that increase the risk and severity of infection in people with diabetes. It is impossible to describe all infections, but specific mention is made of COVID-19, influenza, tuberculosis, skin and urinary tract infections and diabetes-related foot infections. Finally, we describe the principles of treatment as well as prevention through vaccination.

## How infection affects the incidence of diabetes and glucose regulation

**Fig. 1:**
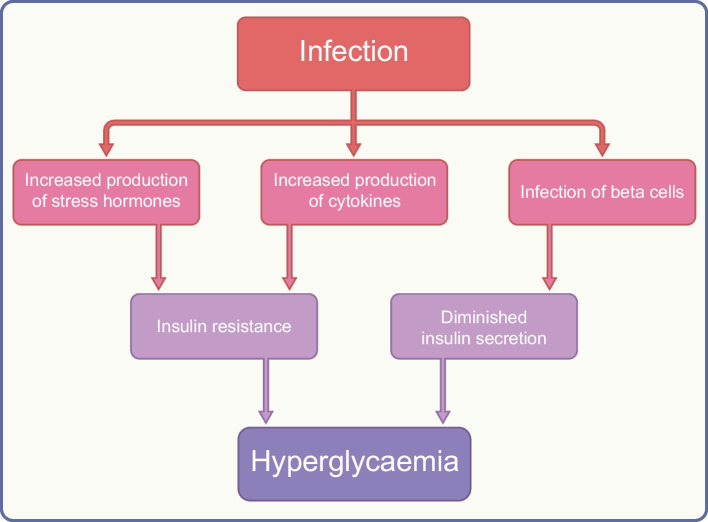
Mechanisms by which infection may worsen glycaemia. Stress hormones include glucagon, growth hormone, catecholamines and glucocorticoid. Cytokines include tumour necrosis factor-α and interleukin-1. This figure is available as part of a downloadable slideset

Infection, and in some cases its treatment, may affect glucose homeostasis through effects on insulin secretion and resistance and increase the risk of diabetes (Fig. 1). Infections are an important predisposing factor for both diabetic ketoacidosis and hyperosmolar hyperglycaemia syndrome, and a search for underlying infection is an important part of the clinical management of acute hyperglycaemic complications.

### Effects on insulin secretion

Multiple viruses have been associated with type 1 diabetes, in particular enteroviruses (especially Coxsackie B1, B4), mumps, rubella and cytomegalovirus (CMV) [14]. Although the literature spans in vitro studies, animal models, human pancreatic tissue and epidemiological studies, the link has not been unequivocally proven in humans [15]. Interest in this area increased following observations that acute and long COVID-19 may be associated with a higher incidence of diabetes. Severe acute respiratory syndrome coronavirus (SARS-CoV)-2 can infect and replicate in human beta cells by gaining entry via ACE-2 receptors [16], but whether this leads to irreversible harm is uncertain. Although early studies suggested an increased incidence of type 1 diabetes [15, 17–21], not all longer term studies have confirmed a significant effect of SARS-CoV-2 [20, 22–28]. Some of the discrepancy between studies may be explained by detection bias as people may be screened for diabetes when presenting with COVID-19 in the community. By contrast, no differences in risk were found in a hospital study, where there were similar opportunities for detection of previously undiagnosed diabetes [28]. Other social, economic and environmental changes that occurred during the pandemic may provide alternative explanations [19, 25, 27]. The CoviDiab registry (https://COVIDiab.e-dendrite.com) has been established to investigate the extent and characteristics of new-onset, COVID-19-related diabetes and will provide further information on the aetiological role of SARS-CoV-2.

### Effect on insulin resistance

Infection induces a stress reaction, increasing the production of counter-regulatory hormones (glucagon, growth hormone, catecholamine and glucocorticoid) and cytokines such as tumour necrosis factor-α and interleukin-1. This combination antagonises insulin action, leading to a failure to suppress hepatic gluconeogenesis and impaired glucose uptake into skeletal muscle [29].

Several viral infections, including infection with hepatitis C virus (HCV) [30, 31] and HIV [32, 33], have been associated with type 2 diabetes. In the case of HCV infection, the inflammation caused by liver damage and subsequent insulin resistance may contribute to the development of hyperglycaemia, but HCV may directly increase insulin resistance by downregulating insulin receptor substrate-1. Successful eradication of HCV improves insulin sensitivity, decreases insulin requirement and lowers blood glucose levels [34]. In addition to the effect of HIV, its treatment with protease inhibitors predisposes to diabetes. These drugs are associated with impaired lipid homeostasis, lipodystrophy, insulin resistance and, to a lesser extent, impaired insulin secretion and mitochondrial dysfunction [35].

Gingivitis and periodontitis are examples of bacterial infection that predispose to diabetes [36]. These infections are associated with local and systemic inflammatory responses that may adversely affect glycaemic levels [37] and increase the risk of diabetes [38, 39]. Treating gingivitis and periodontitis by mechanical removal of dental biofilm and calculus reduces inflammation; meta-analyses of short-duration studies indicate that HbA_1c_ and fasting plasma glucose improve by approximately 3–6 mmol/mol (0.3–0.7%) and 0.5–0.8 mmol/l, respectively, after 3 to 6 months [40–42].

## How diabetes affects the incidence and outcomes of infection

The effect of diabetes on infection has been examined in large population-based cohorts [4–8]. Although studies varied in methods used to capture infection, with some studies reporting hospitalisation for infection and others relying on outpatient clinic codes or prescription of antimicrobial therapy, most studies have reported significant risk associations of diabetes with infection independent of comorbid conditions and other confounding factors. Compared with the general population, people with diabetes have a two- to fourfold increased risk of infection-related hospitalisation and a 1.5-fold increased risk of infection presenting in an outpatient setting [7, 8, 43]. By infection type, the risks are the most pronounced for kidney infection (3.0- to 4.9-fold), osteomyelitis (4.4- to 15.7-fold) and foot infection (6.0- to 14.7-fold), but also increased for pneumonia, influenza, tuberculosis, skin infection, surgical site infection and general sepsis [4, 5, 7, 8, 44].

Diabetes confers a worse outcome from infection, with the most notable example being a twofold higher rate of death from COVID-19 [45]. The risk differential for infection is greater in younger vs older people [4], but seems unaffected by ethnicity [43]. Impaired glucose tolerance is also associated with a higher incidence of infection although the magnitude of the risk is smaller than for diabetes [43, 46].

Where studies considered infection rates in type 1 diabetes and type 2 diabetes separately, the risk ratios were generally higher for type 1 diabetes than type 2 diabetes [6, 47]. In a retrospective UK primary care cohort study including 102,493 people, the incidence rate ratio for hospitalisation related to any infection was 3.7 for type 1 diabetes and 1.9 for type 2 diabetes, and death from infection was increased 7.7-fold in type 1 diabetes and 1.9-fold for type 2 diabetes, relative to adults without diabetes. An Australian study reported a 5.8-fold increase for death from pneumonia, 29.6-fold for osteomyelitis and 9.9-fold for sepsis in people with type 1 diabetes compared with those without diabetes [47].

**Fig. 2:**
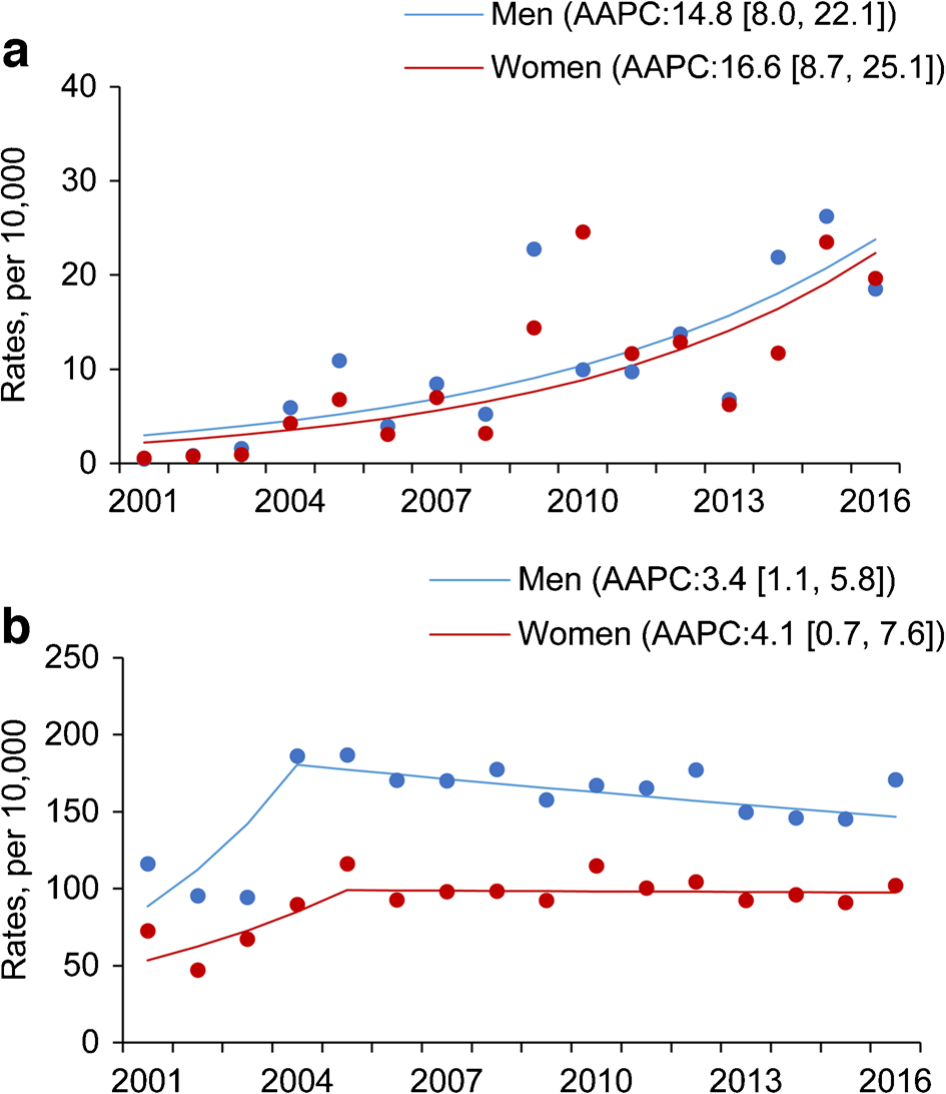
Age-standardised rates of hospitalisation (per 10,000 people) for influenza (**a**) and community-acquired pneumonia (**b**) in men (blue) and women (red) with diabetes in Hong Kong between 2001 and 2016 [4]. Similar trends have also been observed in the USA [7]. Data for average annual percentage change (AAPC) with 95% CI are also shown. This figure has been reproduced from [4] with permission from Springer Nature. This figure is available as part of a downloadable slideset

In contemporary studies examining trends of infection, the rates of infection-related hospitalisation have either remained unchanged or fluctuated in the last two decades. In both the USA and Hong Kong, annualised rates of hospitalisation for influenza showed a rising trend in people with and without diabetes, which may be partly explained by more frequent diagnostic testing leading to better case ascertainment (Fig. 2) [4, 7]. In the USA, hospitalisation for pneumonia decreased in the latter half of the observation period in the general population, while remaining static in people with diabetes, similar to what was observed in Hong Kong [4, 7]. The reasons for these divergent trends of pneumonia between people with and without diabetes are unclear. The sex disparity in the rates of pneumonia in people with diabetes has also been seen in the general population and is likely due to biological differences, such as hormonal cycles and cellular immune-mediated responses, and psychosocial reasons, leading to delayed presentation by men [48]. These observations are worrying when contrasted against decreasing trends of other major clinical events such as cardiovascular disease and lower extremity amputation. Hyperglycaemia and obesity are both risk factors for infection, but there is a lack of consistency between reports about the relationship between HbA_1c_ and infection. Nevertheless, there is a tendency towards more severe infections, especially tuberculosis and kidney infections, with higher HbA_1c_.

## Mechanisms to explain the association of diabetes and infection

**Fig. 3:**
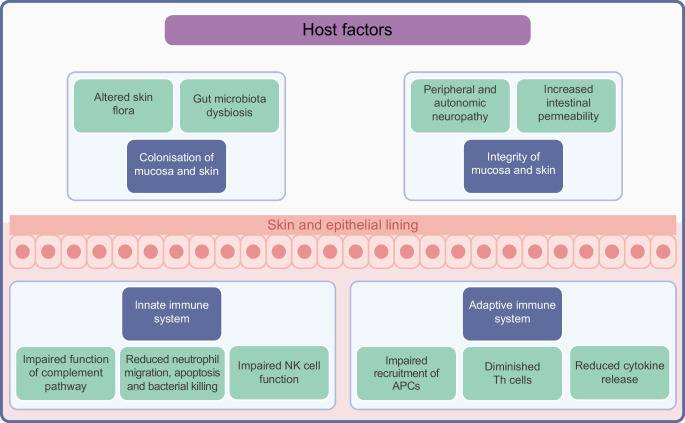
Mechanisms that increase the risk of infection in people with diabetes. Diabetes and its complications impair both innate and adaptive immune systems. The green boxes illustrate how host factors associated with diabetes can affect aspects of the immune system (shown in blue boxes). Some of these host factors affect the likelihood of infection (see processes shown above the skin and epithelial lining) and some affect the response to an infection (see processes shown below the skin and epithelial lining). APC, antigen-presenting cells; NK, natural killer. This figure is available as part of a downloadable slideset

The mechanisms by which diabetes increases the risk of infection can be broadly divided into host factors and organism-specific factors (Fig. 3). Some of the organism-specific factors will be covered in later sections, but in this section we consider the following host factors: the impact of hyperglycaemia on the immune response; vascular insufficiency; sensory peripheral and autonomic neuropathy; skin and mucosal colonisation with pathogens; and increased intestinal permeability and gut-microbiota dysbiosis.

### Impact of the immune response due to hyperglycaemia

Hyperglycaemia has deleterious effects on the innate immune response and adaptive immunity, both of which contribute towards the increased risk of different infections in individuals affected by diabetes.

#### Impairment and modulation of the innate immune system

The innate immune system is often considered the first line of defence of any organism against potential pathogens and infections, even in the absence of prior encounter with the pathogens, for example, in neonates. However, it is also required for the subsequent development of the adaptive response to pathogens encountered, mediated by the expansion of specific clones of B and T lymphocytes. The innate immune response works by having a system and group of proteins and phagocytic cells that can recognise certain conserved features of pathogens, thereby acting against pathogens as soon as they come into contact with the body.

The skin and epithelial lining represent an important part of the innate immune system, and a key barrier against infection. Diabetes increases the risk of different skin lesions and ulcers, for example, diabetes-related foot ulcers, which breach this basic defence and increase the risk of infections. Furthermore, recent studies suggest hyperglycaemia disrupts intestinal barrier function, as well as reprogrammes intestinal epithelial cells, thereby increasing the risk of enteric infections [49].

Diabetes and hyperglycaemia represent states of chronic inflammation that are associated with activation of several components of the innate immune system, including the complement pathway, as well as increased production of several cytokines [50]. The complement pathway is a key component of the innate immune system, with C3 being the central component, and activation to C3b is essential for bacterial opsonisation and subsequent destruction by the membrane attack complex (MAC). While circulating C3 and C4 levels are elevated in people with diabetes, hyperglycaemia leads to an altered structure of C3 and inhibits C3-mediated complement effectors, leading to inhibited immune control of bacterial infections [51]. Diabetes is associated with increased production of reactive oxygen species (ROS), increased release of proinflammatory cytokines and elevated formation of neutrophil extracellular trap (NET). However, it is also linked to reduced neutrophil migration, lower levels of apoptosis, impaired intracellular ROS production and reduced bacterial killing [52]. Natural killer (NK) cells, another key part of the innate immune system, are also impaired in individuals with diabetes, in particular those with long duration of type 1 diabetes [53].

#### Impact of diabetes on the adaptive immune response

Diabetes and hyperglycaemia worsen the adaptive immune response by impairing recruitment and function of antigen-presenting cells (APCs), which results in reduced frequency of T helper (Th)1, Th2 and Th17 cells and the release of cytokines. These, in turn, impact the inflammatory response to encountered pathogens, and contribute to increased risk of infections such as tuberculosis [54]. Interestingly, some of these biological changes are also observed in states of intermediate hyperglycaemia, which highlights the significant role of hyperglycaemia in mediating the risk of infection [55].

#### Other host-specific factors

Several other host-specific factors, notably peripheral vascular disease, peripheral neuropathy increasing the risk of trauma, autonomic neuropathy, skin colonisation with pathogens and the impact of hyperglycaemia itself, combine to contribute to increased risk and severity of foot ulcers in diabetes. There is also increasing interest in the relationship between diabetes, hyperglycaemia and gut-microbiota dysbiosis. In addition to this being considered an important contributor to the metabolic dysfunction and insulin resistance [56], the microbiome contributes to the increased risk of enteric and other systemic infections in diabetes [49, 57].

## Specific infections associated with diabetes

### Respiratory tract infections

#### Community-acquired pneumonia

People with type 2 diabetes have a 1.3- to 2.6-fold higher risk of community-acquired pneumonia and the risks correlate with glycaemic levels [4, 7, 58, 59]. Mortality rates from pneumonia are also increased with diabetes and, in some populations, pneumonia has overtaken cardiovascular disease and cancer as the most common cause of death. Aside from *Streptococcus pneumoniae*, *Staphylococcus aureus* and Gram-negative organisms such as *Klebsiella pneumoniae* are common pathogens leading to lower respiratory tract infections in people with diabetes. In the case of *S. aureus* pneumonia, this may be attributed to higher rates of nasal carriage in people with diabetes (up to 30%) compared with individuals without diabetes (11%) [60]. Diabetes is the commonest underlying predisposing factor for thoracic empyema, with *Klebsiella* spp. being notable common pathogens, followed by streptococci, *S. aureus* and anaerobes [61].

#### Influenza

Influenza is responsible for half a million deaths globally every year. Diabetes increases the risk of severe disease, defined as the requirement for mechanical ventilation, admission to the intensive care unit (ICU) or in-hospital death in young adults (aged 15–50 years) [62], and overall mortality rates [63] by up to fourfold. Influenza and bacterial pneumonia also increase the risk of acute coronary events, which is sustained for weeks to months after the initial exposure [64]. The reasons are multifactorial, possibly related to systemic inflammation causing activation of inflammatory cells in atherosclerotic plaques, a prothrombotic state triggering coronary thrombosis and increased metabolic demand causing cardiac decompensation. Healthcare professionals should ensure that therapy used in primary or secondary cardiovascular protection, such as aspirin and statins, is not interrupted during the course of the infection. Several studies conducted using real-world databases indicate that the use of renin–angiotensin system inhibitors was associated with reduced hospitalisation and/or death from influenza and pneumonia in people with or without diabetes [65–67]. Other glucose-lowering drugs, for example, metformin, may also lower the risk of pneumonia and related mortality rates but these findings are yet to be verified in large RCTs [68, 69].

#### Tuberculosis

People with diabetes are approximately two to three times more likely to develop active tuberculosis than the general population [70, 71]. The public health impact may be particularly high in low- or middle-income countries or areas at the forefront of the diabetes epidemic and where tuberculosis remains endemic, namely in Africa and South-East Asia [71, 72]. In Hong Kong, the risk differential for tuberculosis between people with and without diabetes has remained unchanged over a 10 year period at two- to threefold for middle-aged people with diabetes, but reaching sevenfold in younger age groups [4]. In India, diabetes accounts for 15% of pulmonary tuberculosis and 20% of smear-positive tuberculosis, with an excess risk of the latter in urban areas [73].

Comorbid tuberculosis and diabetes worsens the outcome of both conditions. Extrapulmonary or unusual manifestations of tuberculosis including cavitating disease and involvement of the lower lobes are commoner in people with diabetes, especially those with persistent hyperglycaemia [74]. People with diabetes have a higher risk of treatment failure, relapse and death during treatment [75]. Likewise, tuberculosis can exacerbate hyperglycaemia through stress mechanisms and people with pre-existing diabetes may require intensification of glucose-lowering therapy until active infection is resolved. In a large US longitudinal study, latent tuberculosis infection was associated with a 20% increased risk of developing type 2 diabetes and the risk did not attenuate with treatment of the tuberculosis infection [76]. The World Health Organization recommends that all people with newly diagnosed active tuberculosis should be screened for diabetes if local resources allow, as prompt initiation of glucose-lowering therapy can potentially improve treatment outcomes [77]. Conversely, screening for tuberculosis in people with diabetes is not routinely recommended except in areas with high prevalence of tuberculosis (prevalence over 100 per 100,000 population), in people with specific symptoms or signs and in those with unexplained deterioration in glycaemic levels.

#### Coronavirus

The interaction between diabetes and COVID-19 has been extensively reviewed in the last 3 years [9]. In common with SARS-CoV and Middle East respiratory syndrome-related coronavirus (MERS-CoV), SARS-CoV-2 is an RNA virus that originated from animals. Similar to previous reports that diabetes was an important predictor of severe disease and death in people infected with SARS-CoV and MERS-CoV [78, 79], studies during the pandemic have indicated that diabetes was associated with adverse outcomes and higher mortality rates from COVID-19, although there is no conclusive evidence that diabetes increases the risk of acquiring the viral infection [9, 80]. Between 13% and 58% of people with COVID-19 requiring ICU admission, and between 17% and 35% of people who died, had pre-existing diabetes. Globally, diabetes contributed to 10% of severe forms of COVID-19 and 17% of COVID-19-related deaths [81]. Glycaemic levels appear to affect the outcome of COVID-19. Some, but not all, studies have reported a positive association between HbA_1c_ before hospitalisation and mortality rates from COVID-19 [9]. However, high blood glucose on admission was most consistently predictive of severe disease, irrespective of diabetes status.

### Urinary tract infection

People with diabetes have an increased risk of urinary tract infection, spanning all levels of severity from asymptomatic bacteriuria to renal and perinephric abscess and severe life-threatening emphysematous forms of cystitis and pyelonephritis. Fungal infections, for example, candiduria, and infection with organisms other than *Escherichia coli* are also more frequent in diabetes [82]. The significance of the increased prevalence of asymptomatic bacteriuria in people with diabetes is uncertain because antibiotic treatment does not influence the progression to symptomatic urinary tract infection or risk of pyelonephritis [82]. Autonomic neuropathy is an important predisposing factor; apart from decreased reflex detrusor activity, people with autonomic neuropathy may have impaired bladder sensation resulting in bladder distension, increased residual urine volume and vesicoureteric reflux [83]. The use of sodium–glucose cotransporter 2 (SGLT-2) inhibitors is associated with increased risk of genital tract infections but not urinary tract infections [84].

### Skin and soft tissue infections

Skin infections including dermatophyte infection, candidal intertrigo, bacterial cellulitis and skin abscess are common in people with diabetes. Cellulitis or skin abscesses may be a manifestation of systemic bacteraemia, and diabetes confers a higher risk of mortality due to septicaemia especially among older people. Predisposing factors include peripheral sensory neuropathy, in particular sudomotor dysfunction, causing dryness of skin, dermatophytosis and microvasculopathy. Group A Streptococcus and *S. aureus* are the main culprits for cellulitis, attributable to skin or mucosal colonisation. People with diabetes are more likely to harbour methicillin-resistant *S. aureus* than methicillin-sensitive *S. aureus*, partly due to more frequent attendance of healthcare facilities which increases their exposure to more virulent strains.

A less common but serious soft tissue infection which occurs predominantly in people with diabetes is necrotising fasciitis characterised by inflammation and destruction of fascia, fat and muscle with mixed bacterial involvement. Up to 60% of cases of Fournier gangrene, a form of necrotising fasciitis affecting the perineum, had comorbid diabetes [85]. Given the high mortality of this condition, clinicians need a high index of suspicion to ensure prompt diagnosis and treatment, including broad-spectrum antibiotics and surgical debridement of the affected tissues.

### Diabetes-related foot infection

Infection complicates 50% of diabetes-related foot ulcers and the risk of infection is increased with recurrent or chronic ulcers. Diagnosis of diabetes-related foot infection is based on the presence of cardinal signs of inflammation, although in people with peripheral sensory neuropathy and peripheral artery disease, these signs may not be as apparent. Although the incidence rates of diabetes-related foot ulcers and amputation have declined in many countries, the rates remain high in certain subgroups such as young people, those of minority or indigenous ethnicity, those with social deprivation and people with mental health conditions [86, 87].

## Principles of treatment

### Prevention

Some infection transmission can be reduced through public health measures, such as better personal hygiene, avoidance of crowded conditions and mask wearing, but awareness of and adherence to these preventive measures is low in the general public and in healthcare workers [88–91]. There is no evidence that intensive blood-glucose lowering and weight reduction decreases infection rates in people with diabetes [92].

Some common infections are preventable through vaccination and international guidelines recommend routine vaccination for adults with diabetes (Table 1), although vaccine uptake varies significantly between countries [93]. The evidence on whether diabetes affects immune response to vaccines is unclear and varied depending on the type of vaccine and the age of the population [62]. Vaccination against seasonal influenza is effective both in reducing the likelihood of acquiring the infection and in averting downstream complications. In a large observational cohort of adults with diabetes in Denmark, influenza vaccination was associated with a 15% reduction in risk of all-cause mortality and 16% reduction in risk of cardiovascular mortality [94]. In a population-based study from Canada, influenza vaccination lowered stroke incidence by 31% in people with diabetes vs 17% in those without diabetes, suggesting that vaccination confers similar, if not larger protection against stroke in people with diabetes [95].

**Table 1:** Recommended vaccination schedule for people with diabetes

Vaccine	Age group	Frequency
COVID-19	All	Based on local guidelines
Hepatitis B	<60 years of age, ≥60 years of age after discussion with healthcare professionals	Two- or three-dose series
Human papilloma virus	≤26 years of age, 27–45 years of age after discussion with healthcare professionals	Three doses over 6 months
Influenza	All	Yearly, live attenuated influenza vaccine not recommended
Pneumococcal	All	All children should receive the pneumococcal conjugate vaccine as part of childhood immunisation programme All children and adults with diabetes should receive one dose of pneumococcal conjugate vaccine and one dose of 23-valent pneumococcal polysaccharide vaccine, at least 1 year apart The type of pneumococcal conjugate vaccine and the schedule should be based on local guidelines
Tetanus, diphtheria and acellular pertussis	All	One dose followed by booster every 10 years
Zoster	≥50 years	Two doses, 2–6 months apart

Based on guidance from the ADA Standards of Care [93] and the UK Health Security Agency [107]. Table adapted from [93]. Copyright and all rights reserved. Material from this publication has been used with the permission of American Diabetes Association

In a study in Israel assessing early antibody response to the BNT162b2 COVID-19 vaccine, adults with diabetes had lower IgG concentration than counterparts without diabetes at 5 weeks after receiving the first vaccine dose [96]. In another study comparing humoral and cell-mediated immunogenicity to a COVID-19 vaccine according to attained HbA_1c_, those with HbA_1c_ levels of 53 mmol/mol (7.0%) or higher at the time of vaccination had a weaker immune response than those with lower HbA_1c_ levels [97]. Furthermore, improvement in glycaemic levels was associated with an increase in both neutralising antibody titres and CD4^+^ T cell cytokines. These observations indicate that optimising glycaemic management could be important in maximising the protective effect of COVID-19 vaccine in people with diabetes. In both RCTs and real-world observational studies, COVID-19 vaccine efficacy against SARS-CoV-2 infection and related complications appeared similar between individuals with and without diabetes [98]. In a study in Hong Kong, three doses of COVID-19 vaccine reduced mortality rates by 95% and hospitalisation and ICU admission by 85% to 95% in people with diabetes, comparable to rates in the general population [99].

### Treatment

Treatment of common infections largely follows the same principles as for the general population. Clinical practice guidelines do not generally recommend using a different antimicrobial regimen or setting a lower threshold for initiation of antimicrobial therapy in people with diabetes, although there are some exceptions. For instance, antiviral therapy with nirmatrelvir with ritonavir (Paxlovid) for COVID-19 should be considered in those with diabetes, on top of supportive care. Viral shedding may be more prolonged following influenza infections in people with diabetes, which may influence decisions regarding use of antiviral therapy [100].

Treatment regimens for both drug-susceptible and drug-resistant tuberculosis are the same for people with and without diabetes. However, as diabetes is associated with higher risk of treatment failure, recurrence and emergence of drug-resistant species, extending the treatment period from 6 months to 9 months is recommended, especially when directly observed therapy is not implemented [101, 102]. Close medication supervision and a lower threshold for drug susceptibility testing should be considered for people with diabetes.

For diabetes-related foot infection, successful treatment requires the concerted effort of an interdisciplinary team with expertise in diabetic foot management [103]. Careful assessment of the severity of infection and the presence of complications, such as osteomyelitis, is important to guide clinicians with regard to the antimicrobial regimen and length of treatment, as well as the need for hospitalisation and orthopaedic and vascular interventions. There have been new advances in wound management such as hyperbaric oxygen therapy, topical oxygen therapy and negative pressure wound therapy, but evidence supporting their role in treatment of foot infection is currently weak.

People with diabetes should be monitored closely for clinical deterioration and other complications as they are more likely to harbour multidrug-resistant pathogens and have worse outcomes from infections. To minimise antimicrobial resistance, the principles of antibiotic stewardship should be judiciously observed. For instance, antimicrobial therapy for asymptomatic bacteriuria does not reduce the rate of symptomatic urinary tract infection or pyelonephritis in people with diabetes and is generally not recommended [104]. Likewise, antimicrobial therapy should not be used in people with uninfected foot ulcers as prophylactic antimicrobial therapy does not improve outcome [103].

Comorbidities and polypharmacy are common in people with diabetes and due caution should be exercised to avoid clinically relevant drug–drug interactions and serious drug toxicities. For instance, the antimicrobial dose may need to be down-titrated in kidney impairment, while administration of nephrotoxic drugs may worsen kidney function in those with underlying chronic kidney disease. Certain antimicrobials (e.g., rifampicin) affect the hepatic metabolism of glucose-lowering drugs (such as sulfonylureas), thus altering glucose-lowering efficacy. Exposure to drugs that affect vision (e.g., ethambutol) or peripheral nerves (e.g., isoniazid) or drugs that are ototoxic (such as aminoglycosides) may be particularly problematic in people who are partially sighted due to diabetic retinopathy or in those who have other disabilities affecting daily living.

Hyperglycaemia during hospital admission for sepsis and other infections predicts a longer hospital stay and increased mortality rates. In a landmark study examining the effects of glycaemic management in people admitted to the ICU, among whom one-fifth had sepsis, lowering of blood glucose to 4.5–6.0 mmol/l did not lead to improved outcomes compared with moderate blood glucose targets of ≤10 mmol/l [105]. Indeed, intensive blood-glucose lowering was associated with more hypoglycaemic episodes and slightly increased mortality rates. It is recommended that SGLT-2 inhibitors should be temporarily withheld in critically ill people, including those with serious infection, because of the potential risk of diabetic ketoacidosis. However, an RCT of individuals with at least one cardiometabolic comorbidity admitted with COVID-19 showed that in-hospital initiation of dapagliflozin had no effect on organ dysfunction or death vs placebo, and did not increase the rates of diabetic ketoacidosis or acute kidney injury [106].

## Conclusion

This review discusses how infection remains an important cause of morbidity and mortality for people with diabetes. The risks of infection and worse outcomes are higher in people with diabetes. In part, this relates to the effect of hyperglycaemia on the body’s defence mechanisms against infection, but other host-specific and pathogen-specific factors play a role. Infections may worsen glycaemic levels or increase the risk of diabetes. Significant gaps in our knowledge about the relationship between diabetes and infection remain. While trends in incidence of other diabetes complications have been well reported in many regions, the epidemiology of infection is less well described. Most studies do not differentiate between type 1 diabetes and type 2 diabetes; consequently, it is uncertain how infection differs between types of diabetes and whether this has any clinical implications for prevention of infection and responses to antimicrobial therapy. The variety in pathogens and sites of infection further complicate research in this area. Lack of consistency in methods used to define infective episodes in epidemiological studies is a challenge. It is unclear whether people with diabetes have reduced immune responses to vaccine and, if so, what factors may modify vaccine effectiveness (e.g., BMI or glycaemia). Major advances in diabetes treatments and technologies have occurred, but it is unknown whether the improved diabetes care translates to reduction in risk of serious infection. The effects of new anti-diabetes drugs on infection risk should be included as study endpoints in future RCTs of glucose-lowering therapies.

### Supplementary Information

Below is the link to the electronic supplementary material.

Supplementary file1 (PPTX 431 KB)

## Abbreviations

HCV: Hepatitis C virus
ICU: Intensive care unit
MERS-CoV: Middle East respiratory syndrome-related coronavirus
ROS: Reactive oxygen species
SARS-CoV: Severe acute respiratory syndrome coronavirus
SGLT-2: Sodium–glucose cotransporter 2
Th: T helper

## Authors’ relationships and activities

RIGH has received honoraria for speaker engagement and conference attendance from Eli Lilly, Novo Nordisk and Rovi. RCWM has received research grants from AstraZeneca, Bayer, Novo Nordisk, Pfizer, Roche Diagnostics (HK) Ltd and Tricida Inc., and consultancy/speaker honoraria from AstraZeneca, Boehringer Ingelheim, Bayer and Merck. All proceeds have been donated to the Chinese University of Hong Kong to support diabetes research. RCWM is a co-founder of GemVCare, a technology start-up initiated with support from the Hong Kong Government Innovation and Technology Commission and its Technology Start-up Support Scheme for Universities (TSSSU). RCWM is a member of the Editorial Board of *Diabetologia*. AOYL has served as a member of advisory panels for Amgen, AstraZeneca, Boehringer Ingelheim and Sanofi, and has received research support from Amgen, the Asia Diabetes Foundation, Bayer, Biogen, Boehringer Ingelheim, Lee’s Pharmaceutical, MSD, Novo Nordisk, Roche, Sanofi, Sugardown Ltd. and Takeda. None of these relationships or activities are related to the content of the current article. CSC declares that there are no relationships or activities that might bias, or be perceived to bias, their work.
