# Supplementary material for: Diabetes and infection: review of the epidemiology, mechanisms and principles of treatment
Source: Diabetologia. 2024 Feb 20;67(7):1168–80. doi: 10.1007/s00125-024-06102-x (PMC11153295; doi:10.1007/s00125-024-06102-x)
Supplement: Supplementary file 1 — Supplementary file1 (PPTX 431 KB) [file 125_2024_6102_MOESM1_ESM.pptx]

## Slide 1
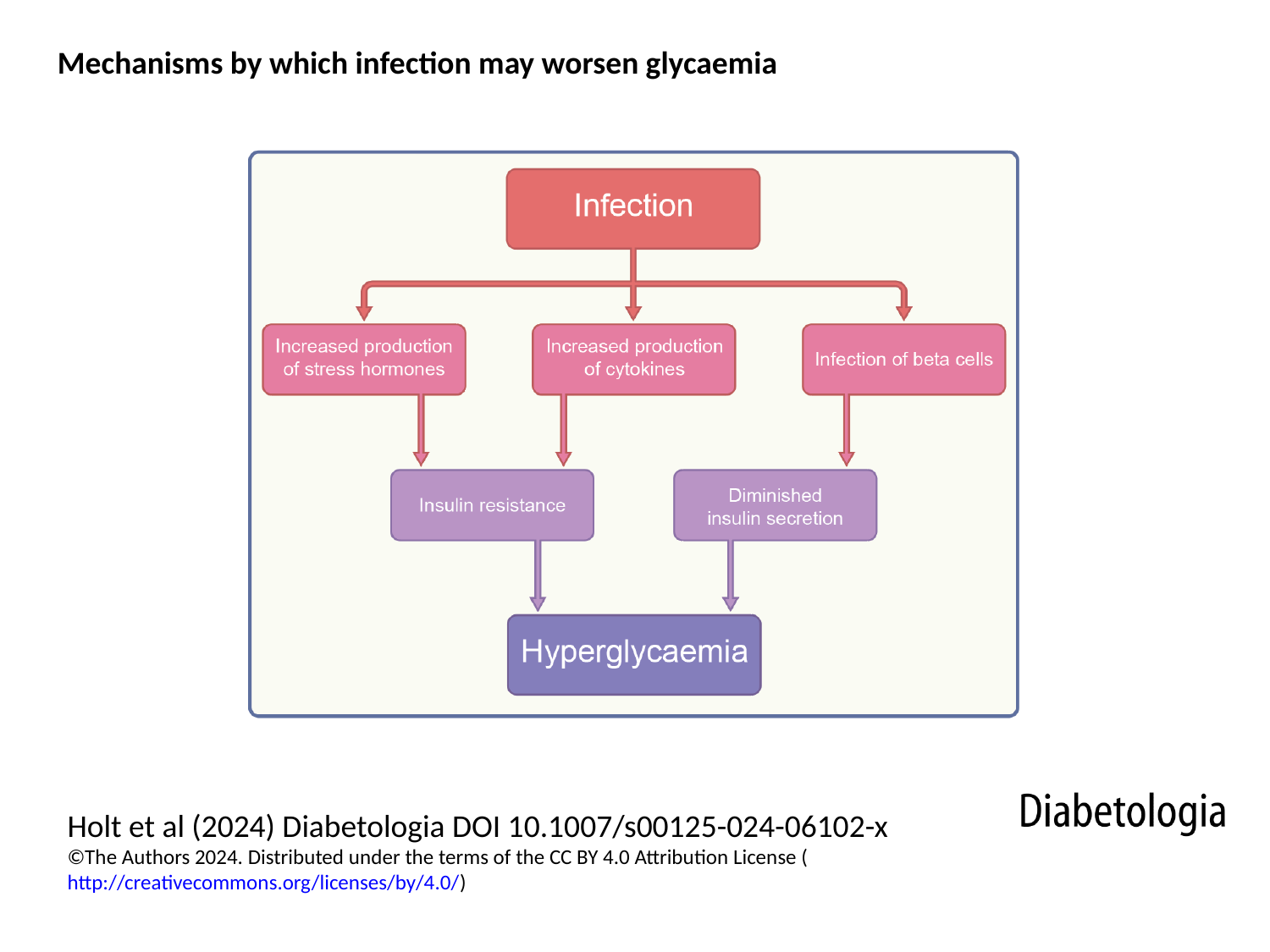

Mechanisms by which infection may worsen glycaemia
Holt et al (2024) Diabetologia DOI 10.1007/s00125-024-06102-x
©The Authors 2024. Distributed under the terms of the CC BY 4.0 Attribution License (http://creativecommons.org/licenses/by/4.0/)

## Slide 2
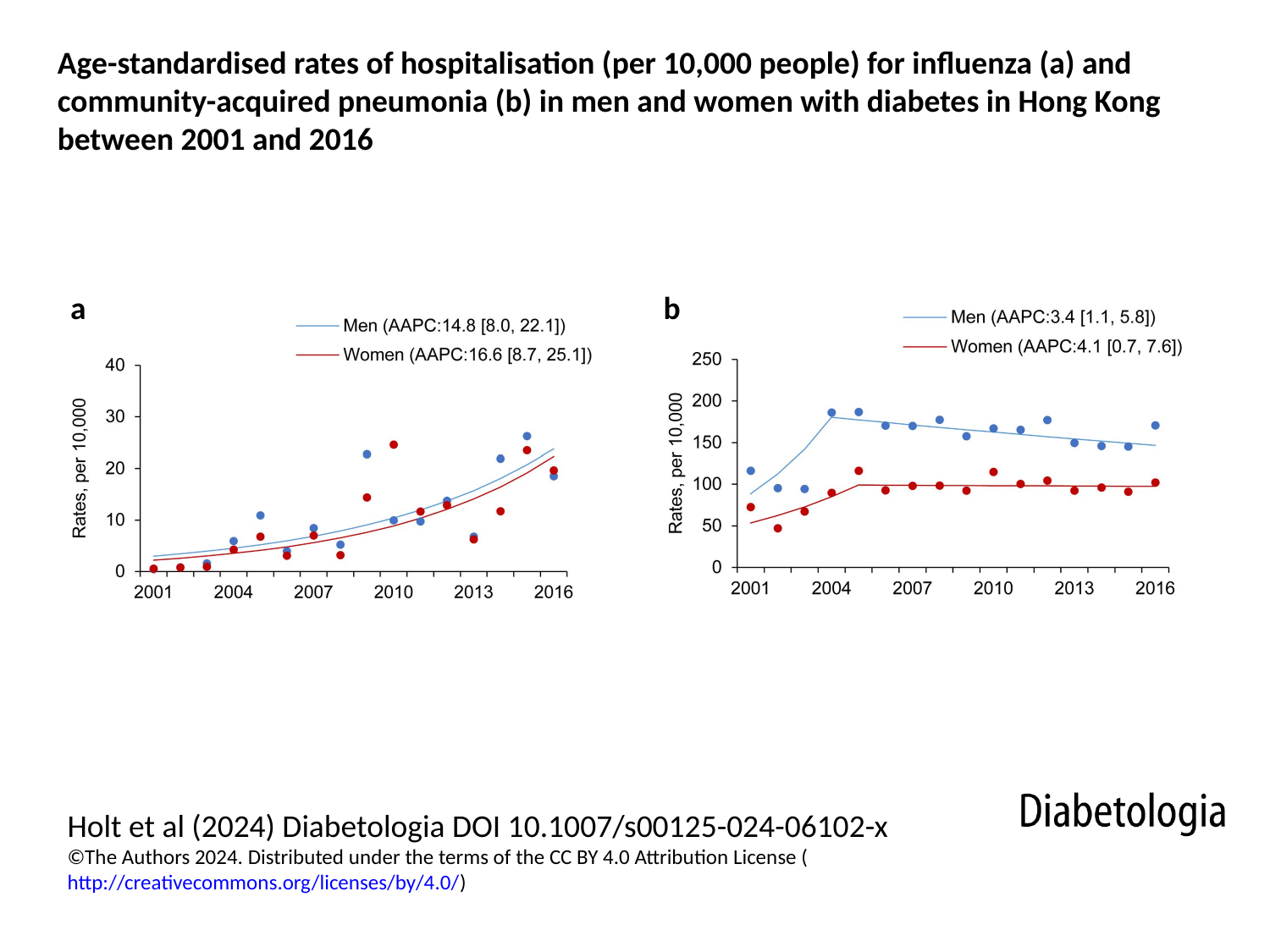

Age-standardised rates of hospitalisation (per 10,000 people) for influenza (a) and community-acquired pneumonia (b) in men and women with diabetes in Hong Kong between 2001 and 2016
a
b
Holt et al (2024) Diabetologia DOI 10.1007/s00125-024-06102-x
©The Authors 2024. Distributed under the terms of the CC BY 4.0 Attribution License (http://creativecommons.org/licenses/by/4.0/)

## Slide 3
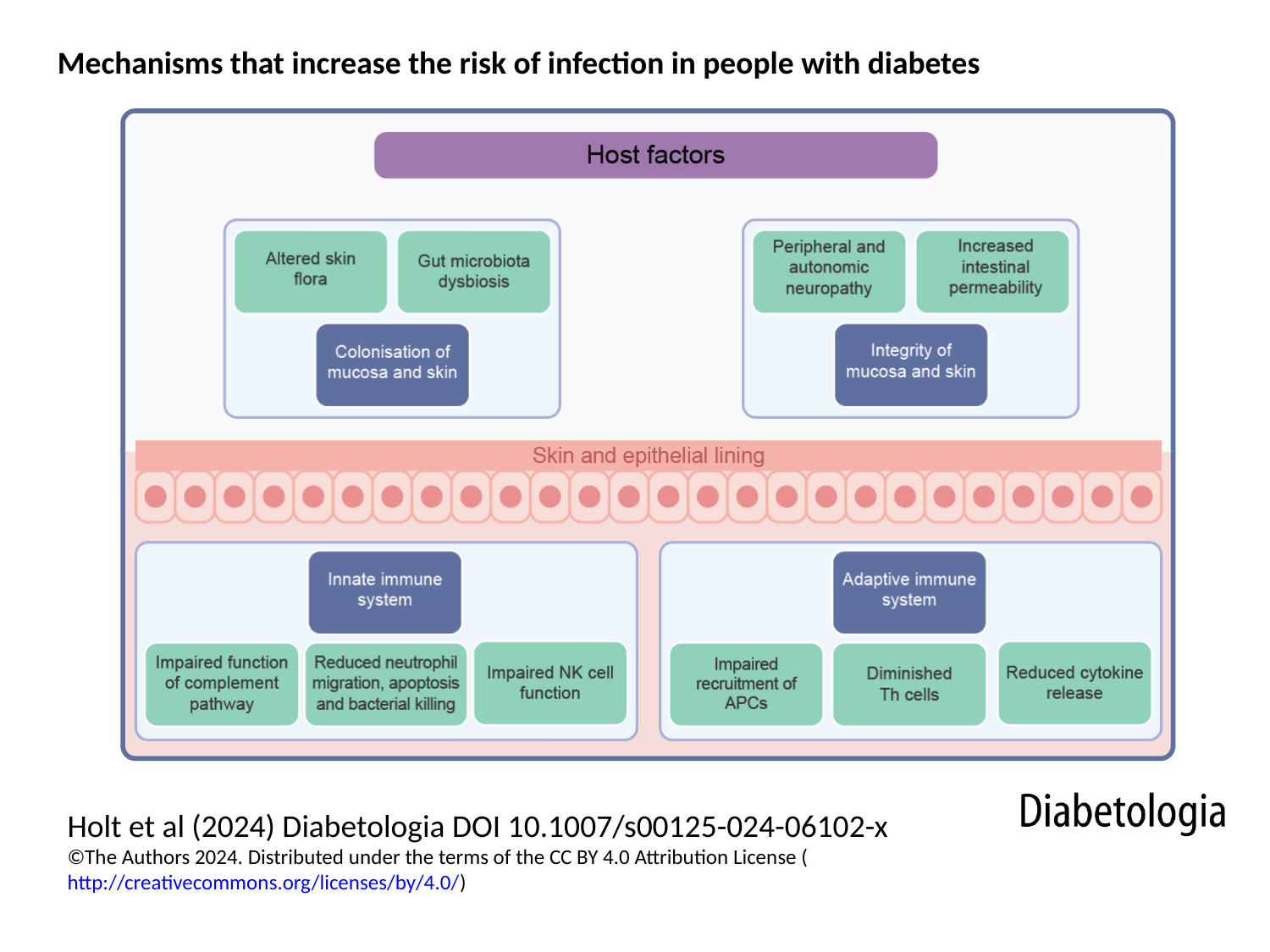

Mechanisms that increase the risk of infection in people with diabetes
Holt et al (2024) Diabetologia DOI 10.1007/s00125-024-06102-x
©The Authors 2024. Distributed under the terms of the CC BY 4.0 Attribution License (http://creativecommons.org/licenses/by/4.0/)
